# Iron-Mediated Homogeneous ICAR ATRP of Methyl Methacrylate under ppm Level Organometallic Catalyst Iron(III) Acetylacetonate

**DOI:** 10.3390/polym8020029

**Published:** 2016-01-26

**Authors:** Jian Wu, Xiaowu Jiang, Lifen Zhang, Zhenping Cheng, Xiulin Zhu

**Affiliations:** Suzhou Key Laboratory of Macromolecular Design and Precision Synthesis, Jiangsu Key Laboratory of Advanced Functional Polymer Design and Application, Department of Polymer Science and Engineering, College of Chemistry, Chemical Engineering and Materials Science, Soochow University, Suzhou 215123, China; wujian_suda@163.com (J.W.); jiang_xiao_wu@163.com (X.J.); xlzhu@suda.edu.cn (X.Z.)

**Keywords:** ICAR ATRP, organometallic catalyst, Fe(acac)_3_

## Abstract

Atom Transfer Radical Polymerization (ATRP) is an important polymerization process in polymer synthesis. However, a typical ATRP system has some drawbacks. For example, it needs a large amount of transition metal catalyst, and it is difficult or expensive to remove the metal catalyst residue in products. In order to reduce the amount of catalyst and considering good biocompatibility and low toxicity of the iron catalyst, in this work, we developed a homogeneous polymerization system of initiators for continuous activator regeneration ATRP (ICAR ATRP) with just a ppm level of iron catalyst. Herein, we used oil-soluble iron (III) acetylacetonate (Fe(acac)_3_) as the organometallic catalyst, 1,1′-azobis (cyclohexanecarbonitrile) (ACHN) with longer half-life period as the thermal initiator, ethyl 2-bromophenylacetate (EBPA) as the initiator, triphenylphosphine (PPh_3_) as the ligand, toluene as the solvent and methyl methacrylate (MMA) as the model monomer. The factors related with the polymerization system, such as concentration of Fe(acac)_3_ and ACHN and polymerization kinetics, were investigated in detail at 90 °C. It was found that a polymer with an acceptable molecular weight distribution (*M*_w_/*M*_n_ = 1.43 at 45.9% of monomer conversion) could be obtained even with 1 ppm of Fe(acac)_3_, making it needless to remove the residual metal in the resultant polymers, which makes such an ICAR ATRP process much more industrially attractive. The “living” features of this polymerization system were further confirmed by chain-extension experiment.

## 1. Introduction

Reversible deactivation radical polymerization (RDRP) [1,2,3,4,5,6,7] including initiator-transfer agent-terminator (Iniferter) [8,9,10,11], nitroxide-mediated polymerization (NMP) [12,13,14,15,16,17,18], atom transfer radical polymerization (ATRP) or metal-catalyzed living radical polymerization [19,20,21,22,23,24,25,26,27,28,29,30,31,32,33,34,35,36,37,38,39,40,41,42,43] and reversible addition−fragmentation chain transfer polymerization (RAFT) [44,45,46,47,48,49,50,51,52,53,54,55] has been used to design and synthesize various polymeric structure and architectures extensively. Among those methods, ATRP is the most widely used method and has been used to produce different topological polymers, such as star, brush, block and hyperbranched polymers [56,57,58,59]. On the other hand, it is well known that some shortages of ATRP also exists, such as the large amount of metal catalyst residues in products. In 2007, Matyjaszewski’s group found that in the polymerization systems (e.g., MMA/EBPA/Cu(II)/tris(2-pyridylmethyl)amine (TPMA)/azobis(isobutyronitrile) (AIBN)), reducing the amount of catalyst (<50 ppm) while increasing the amount of traditional radical initiator (such as [AIBN]_0_/[Cu]_0_ > 10) could control the polymerization systems quite well. They called this method initiators for continuous activator regeneration atom transfer radical polymerization (ICAR ATRP) [60,61,62,63,64,65]. Actually, the most important advantage of ICAR ATRP [60,61,62,63,64,65] over normal ATRP is that the desired amount of catalyst is significantly reduced because the continuously produced free radicals can reduce the high oxidation transition metal complexes so that the catalyst can remain active during the polymerization process.

There have been many catalysts which can be used in ICAR ATRP, such as Cu [66,67], Ru [68], and Fe [69,70]. However, most of them are poisonous and expensive. Therefore the application of many catalysts is limited in large-scale industrial production especially in the field of biomedical and electrical materials. Because of the low toxicity and good biocompatibility of iron salt, the iron catalytic systems are more and more popular in academic research community since the Sawamoto’s group reported the controlled polymerization system catalyzed by FeCl_2_/PPh_3_ [71]. The common ligands for iron salt are nitrogen-containing ligands [72,73,74,75,76,77,78,79], phosphorus-containing ligands [80,81,82,83,84,85,86,87,88], organic acids [89,90,91,92,93] and polyethylene glycols [94]. Furthermore, the application of many catalysts (e.g., inorganic transition metal catalysts) is limited because of their solubility in oil-soluble reaction systems. In 2014, an organic transition metal catalyst iron(III) acetylacetonate (Fe(acac)_3_) was employed as the catalyst of activators generated by electron transfer for ATRP (AGET ATRP) of methyl methacrylate (MMA) using ascorbic acid (AsAc) as reducing agent for the first time [95]. However, the concentration of iron catalyst is relatively high and the polymerization system was heterogeneous due to the use of polar reducing agent ascorbic acid.

Based on these problems, we try to use an organic iron salt as the catalyst to set up a homogeneous iron-mediated ICAR ATRP suitable for oil-soluble polymerization system, which can be controllable when the amount of catalyst is reduced to ppm level. It should be noted that azobisisobutyronitrile (AIBN) is used as the thermal initiator in an ICAR ATRP system usually. However, we notice that 1,1′-azobis cyclohexanecarbonitrile (ACHN) with a longer half-life period (10 h at 88 °C) [96] is a more thermally stable azo-initiator than AIBN (10 h at 65 °C), which facilitates to conduct ICAR ATRP at a relatively higher temperature. Herein, we used ACHN as the thermal initiator to establish an iron-mediated homogeneous ICAR ATRP system using oil-soluble Fe(acac)_3_ as the organometallic catalyst and PPh_3_ as the ligand. This polymerization system provided an efficient homogeneous polymerization of MMA under ppm level of iron catalyst at 90 °C.

## 2. Experimental Section

### 2.1. Materials

The monomer, methyl methacrylate (MMA, +99%), was purchased from Shanghai Chemical Reagents Co. Ltd. (Shanghai, China). It was purified via removing the inhibitor by passing through a short neutral alumina column before use. Azobis(isobutyronitrile) (AIBN), which was purchased from Shanghai Chemical Reagents Co. Ltd. and 1,1′-azobis cyclohexanecarbonitrile (ACHN, 98%, Aldrich, Shanghai, China) were purified by recrystallization from ethanol. The following materials were used as received. These materials are ethyl 2-bromophenylacetate (EBPA, 97%) purchased from J&K Scientific Ltd. (Beijing, China), triphenylphosphine (PPh_3_, 98%) purchased from Energy chemical (Shanghai, China), ethyl 2-bromo-2-methylpropionate (EBiB, 98%) purchased from Acrso, tetrahydrofuran (THF, analytical reagent) purchased from Nanjing Chemical Reagent Co. Ltd. (Nanjing, China), toluene (analytical reagent) purchased from Chinasun Specialty Products Co. Ltd. (Changshu, China) and iron(III) acetylacetonate (Fe(acac)_3_, 99.95%) purchased from Aldrich (Shanghai, China).

### 2.2. General Procedure for ICAR ATRP of MMA

A typical solution polymerization procedure for the molar ratio of [MMA]_0_:[EBPA]_0_:[Fe(acac)_3_]_0_:[PPh_3_]_0_:[ACHN]_0_ = 200:1:0.03:0.3:1 is as follows. A homogeneous mixture was obtained by adding Fe(acac)_3_ (0.51 mg), PPh_3_ (3.7 mg), MMA (1.0 mL), EBPA (8.3 μL), ACHN (8.0 mg), toluene (1.0 mL) and a magnetic stirring bar to a clean ampoule. The mixture was thoroughly bubbled with argon for 20 min to eliminate the dissolved oxygen in the reaction system, and then flame-sealed; afterwards it was transferred into an oil bath held by a magnetic stirring apparatus at the desired temperature (90 °C) to polymerize under stirring. After the desired polymerization time, the ampoule was cooled by immersing it into iced water. Afterwards, it was opened and the contents were dissolved in THF (~2 mL), and precipitated into a large amount of methanol (~200 mL). The polymer obtained by filtration was dried under vacuum until constant weight at 35 °C. The monomer conversion was determined gravimetrically.

### 2.3. Chain Extension of PMMA

A predetermined quantity of PMMA obtained by ICAR ATRP of MMA was added to a clean ampoule, and then the determined quantities of MMA (0.2 mL), toluene (1.0 mL), Fe(acac)_3_, PPh_3_ and ACHN were added. The mixture was thoroughly bubbled with argon for 20 min to eliminate the dissolved oxygen in the reaction system, and then flame-sealed; afterwards it was transferred into an oil bath held by a thermostat at the desired temperature (90 °C) to polymerize under stirring. The rest of the procedures were the same as that for the polymerization of MMA described above.

### 2.4. Characterization

The number-average molecular weight (*M*_n,GPC_) and molecular weight distribution (*M*_w_/*M*_n_) values of the resultant polymers were determined by a TOSOH HLC-8320 (Tosoh, Japan) gel permeation chromatograph (GPC) equipped with a refractive-index detector (TOSOH), using TSKgel guardcolumn SuperMP-N (4.6 mm× 20 mm) and two TSKgel SupermultiporeHZ-N (4.6 mm× 150 mm) with measurable molecular weight ranging from 5 × 10^2^ to 5 × 10^5^ g/mol. THF was used as the eluent at a flow rate of 0.35 mL/min and 40 °C. GPC samples were injected using a TOSOH plus autosampler and calibrated with PMMA standards purchased from TOSOH. ^1^H NMR spectrum of the obtained polymer was recorded on Bruker 300 (Bruker, Coventry, UK) MHz nuclear magnetic resonance (NMR) instrument using DMSO-d_6_ as the solvent and tetramethylsilane (TMS) as an internal standard at ambient temperature.

## 3. Results and Discussion

### 3.1. Effect of Type of Initiator and Solvent on Polymerization of MMA

Firstly, we selected two kinds of ATRP initiators EBPA and EBiB to conduct ICAR ATRP while using ACHN as the thermal initiator. From Table 1, it can be seen that when EBPA (Entries 1, 2 in Table 1) was used as the ATRP initiator, the results prove the better efficiency than EBiB (Entries 3, 4 in Table 1). Specifically, the *M*_n,GPC_ values are much closer to their corresponding *M*_n,th_ ones and narrower molecular weight distributions in the case of EBPA. Therefore, in consideration of significant effect from initiators on polymerization controllability and the facility of the chain-end characterization, the active EBPA, with a phenyl group as the internal standard, was selected as a more appropriate and efficient initiator for the ICAR ATRP of MMA for the following investigation. In addition, in order to study the effect of solvent on the polymerization, we used three different solvents to conduct the polymerization. As is shown in Table 1, the molecular weight distribution of the polymer obtained by using ethanol (Entry 6 in Table 1) as the solvent is broader than using toluene (Entries 1, 2, 5 in Table 1). On the other hand, the polymerization rate using toluene (Entry 5 in Table 1) as the solvent is faster than ethyl acetate (Entry 7 in Table 1). Therefore, toluene was used as the solvent for the further investigation.

**Table 1:** Effect of different initiators and solvents on initiators for continuous activator regeneration (ICAR) Atom Transfer Radical Polymerization (ATRP) of methyl methacrylate (MMA).

Entry	R	T (h)	Conv. (%)	*M*_n,th_^f^ (g/mol)	*M*_n,GPC_ (g/mol)	*M*_w_*/M*_n_
1 ^a^	200:1:0.02:0.3:1	3.5	64.0	12,800	17,000	1.18
2 ^a^	200:1:0.015:0.3:1	3.5	54.7	10,900	15,900	1.19
3 ^b^	200:1:0.02:0.3:1	1.5	43.7	8800	96,900	1.56
4 ^b^	200:1:0.01:0.3:1	1.5	43.5	8700	96,500	1.46
5 ^c^	200:1:0.02:0.3:1	3	56.0	11,200	16,500	1.17
6 ^d^	200:1:0.02:0.3:1	3	53.1	10,600	24,600	1.40
7 ^e^	200:1:0.02:0.3:1	3	36.1	7200	9500	1.18

Polymerization conditions: R = [MMA]_0_:[I]_0_:[Fe(acac)_3_]_0_:[PPh_3_]_0_:[ACHN]_0_, *V*_MMA_ = 1.0 mL; ^a,b,c^ *V*_toluene_ = 1.0 mL; ^d^ *V*_ethanol_ = 1.0 mL; ^e^ *V*_ethyl acetate_ = 1.0 mL, T = 90 °C; ^a,c,d,e^ I = EBPA; ^b^ I = EBiB; ^f^ *M_n,th_* = ([M]_0_/[I]_0_) × *M*_n,MMA_ × conv.%.

### 3.2. Comparison of Using AIBN and ACHN as the Thermal Initiator

In ICAR ATRP, we usually use AIBN as the azo-initiator; in this work, we used ACHN as the thermal initiator and compared the polymerization behaviors of these two azo-initiators. As shown in Table 2, when the concentration of catalyst is high enough, the molecular weight distributions of the resultant polymers in both azo-initiator cases (*M*_w_*/M*_n_ ≤ 1.30), but *M*_n,GPC_ values are closer to *M*_n,th_ when using ACHN (Entries 1, 2, 4 and 5 in Table 2). However, when the concentration of catalyst is much low ([Fe(acac)_3_] = 5 ppm, Entry 3 in Table 2), the molecular weight distribution (*M*_w_/*M*_n_ = 1.42 in the case of AIBN) is broader than that of using ACHN (*M*_w_/*M*_n_ = 1.27) (Entry 6 in Table 2). Therefore, ACHN is more suitable for this polymerization system. It is contributed to the fact that ACHN has a longer half-life period than AIBN, which facilitates to produce free radicals to reduce Fe(III) species continuously.

**Table 2:** Effect of type of azo-initiators on ICAR ATRP of MMA.

Entry	R	T (h)	Conv. (%)	*M*_n,th_^c^ (g/mol)	*M*_n,GPC_ (g/mol)	*M*_w_*/M*_n_
1 ^a^	200:1:0.02:0.3:1	2.5	40.8	8200	14,900	1.16
2 ^a^	200:1:0.01:0.3:1	2.5	37.6	7500	17,100	1.22
3 ^a^	200:1:0.001:0.3:1	2	58.2	11,600	24,200	1.42
4 ^b^	200:1:0.02:0.3:1	3.5	64.0	12,800	17,000	1.18
5 ^b^	200:1:0.015:0.3:1	3.5	54.7	10,900	15,900	1.19
6 ^b^	200:1:0.001:0.3:1	3	50.1	10,000	21,100	1.27

Polymerization conditions: R = [MMA]_0_:[EBPA]_0_:[Fe(acac)_3_]_0_:[PPh_3_]_0_:[RA]_0_, *V*_MMA_ = 1.0 mL, *V*_toluene_ = 1.0 mL; ^a^ RA = AIBN, T = 70 °C; ^b^ RA = ACHN, T = 90 °C; ^c^ *M*_n,th_ = ([M]_0_/[I]_0_) × *M*_n,MMA_ × conv.%.

### 3.3. Effect of Concentration of Iron Catalyst on Polymerization of MMA

From the discussion above, we can see that using ACHN as the azo-initiator can achieve a better control of the polymerization. In order to examine the effect of the concentration of iron catalyst on the polymerization, we keep the same molar ratio of [MMA]_0_:[EBPA]_0_:[PPh_3_]_0:_[ACHN]_0_ to conduct polymerizations under different iron catalyst concentrations. From Table 3, it can be seen that the amount of iron catalyst had a little influence on the reaction rate and as the iron catalyst concentration increased, the molecular weight distributions become narrower, which indicated that the control of the polymerization system is better (Entry 1–6 in Table 3). This is probably because the ability of the catalytic system deactivating chain-growth radical improved as the concentration of iron catalyst increased. Therefore, the free radical concentration decreased and the polymerization system could be well controlled [97]. It is important and worth noting that even though the concentration of iron catalyst decreased to 1 ppm (Entry 9 in Table 3), the polymerization still took place in a controlled manner (*M*_w_/*M*_n_ = 1.43), which indicated that this iron-mediated ICAR ATRP system had a high catalytic activity. It is worth mentioning that the deviation of *M*_n,th_ and *M*_n,GPC_ increased with the decreasing amount of iron catalyst. This may contribute to the fact that the deactivation reaction is less controlled in the beginning of ATRP (in particular due to the lack of halide in Fe(acac)_3_), but the situation subsequently improves toward moderate and high degrees of monomer conversion.

**Table 3:** Effect of the concentration of the catalyst on ICAR ATRP of MMA

Entry	Catalyst Concentration (ppm)	T (h)	Conv. (%)	*M*_n,th_^a^ (g/mol)	*M*_n,GPC_ (g/mol)	*M*_w_/*M*_n_
1	150	2	27.4	5500	10,600	1.17
2	150	4	66.5	13,300	19,500	1.13
3	100	2	41.0	8200	15,700	1.20
4	100	4	68.0	13,600	19,000	1.14
5	75	2	30.6	7100	11,800	1.23
6	75	4	63.0	12,600	17,400	1.18
7	5	3	50.1	10,000	21,140	1.27
8	2.5	2	45.2	9000	32,200	1.39
9	1	2	45.9	9200	43,300	1.43

Polymerization conditions: R = [MMA]_0_:[EBPA]_0_:[Fe(acac)_3_]_0_:[PPh_3_]_0_: [ACHN]_0_ = 200:1:*x*:0.3:1 (*x* = 0.03 (150 ppm), 0.02 (100 ppm), 0.015 (75 ppm), 0.001 (5 ppm), 0.0005 (2.5 ppm), and 0.0002 (1.0 ppm)), *V*_MMA_ = 1.0 mL, *V*_toluene_ = 1.0 mL, T = 90 °C; ^a^ *M*_n,th_ = ([M]_0_/[I]_0_) × *M*_n,MMA_ × conv.%.

### 3.4. Effect of Concentration of ACHN on Polymerization of MMA

In order to examine the effect of the concentration of ACHN on the polymerization, we kept the molar ratio of [MMA]_0_:[EBPA]_0_:[Fe(acac)_3_]_0_:[PPh_3_]_0_ constant and changed the amount of ACHN constantly to carry out the polymerization of MMA. As shown in Table 4, when [ACHN]_0_/[EBPA]_0_ = 0.1 (Entry 1 in Table 4), no polymers were obtained after 2.5 h. However, as the concentration of ACHN increased, the monomer conversion increased under the same polymerization time (2.5 h) as expected by the mechanism of ICAR ATRP [60,61,62,63,64,65,69]. When [ACHN]_0_/[EBPA]_0_ = 2.5 (Entry 6 in Table 4), the monomer conversion could achieve 73.5% after 2.5 h with a controlled molecular weight distribution (*M*_w_/*M*_n_ = 1.21).

**Table 4:** Effect of the concentration of 1,1′-azobis (cyclohexanecarbonitrile) (ACHN) on ICAR ATRP of MMA.

Entry	*x*	Conv. (%)	*M*_n,th_^a^ (g/mol)	*M*_n,GPC_ (g/mol)	*M*_w_/*M*_n_
1	0.1	–	–	–	–
2	0.5	24.4	4900	10,400	1.19
3	1.0	48.0	9600	16,400	1.30
4	1.5	62.6	12,500	16,700	1.17
5	2.0	63.2	12,600	18,400	1.17
6	2.5	73.5	14,700	19,900	1.21

Polymerization conditions: R = [MMA]_0_:[EBPA]_0_:[Fe(acac)_3_]_0_:[PPh_3_]_0_: [ACHN]_0_ = 200:1:0.02:0.3:*x* (*x* = 0.1, 0.5, 1.0, 1.5, 2.0 and 2.5), *V*_MMA_ = 1.0 mL, *V*_toluene_ = 1.0 mL, T = 90 °C, polymerization time = 2.5 h; ^a^ *M*_n,th_ = ([M]_0_/[I]_0_) × *M*_n,MMA_ × conv.%.

### 3.5. Variation of the Target Degree of Polymerization

In order to examine the effect of polymerization degree, the polymerizations of MMA were conducted with various molar ratios of monomer. We keep the amount of solvent, catalyst, and initiator constant, and change the amount of monomer to carry out the polymerization. When DP = 300, the monomer conversion is 43.9% after 2.5 h (*M*_w_/*M*_n_ = 1.15) (Entry 1 in Table 5). When DP = 1000, the monomer conversion decreased to 36.3% (Entry 1 in Table 5). This phenomenon showed that the polymerization rate became slower as the absolute concentrations of the thermal initiator is decreased and the molecular weight distributions changed to a little broader as the amount of monomer increased, but the molecular weight distribution (*M*_w_/*M*_n_ = 1.24) was still acceptable and *M*_n,GPC_ (*M*_n,GPC_ = 43,300 g/mol) was closer to *M*_n,th_ (*M*_n,th_ = 36,300 g/mol). Therefore, the activity of this polymerization system was much high even under a high target polymerization degree.

**Table 5:** Effect of molar ratios of monomer on ICAR ATRP of MMA

Entry	R	Conv.(%)	^a^ *M*_n,th_ (g/mol )	*M*_n,GPC_ (g/mol )	*M*_w_/*M*_n_
1	300:1:0.02:0.3:1	43.9	13,200	21,500	1.15
2	400:1:0.02:0.3:1	43.0	17,200	24,900	1.16
3	800:1:0.02:0.3:1	39.6	31,700	41,100	1.20
4	1000:1:0.02:0.3:1	36.3	36,300	43,300	1.24

Polymerization conditions: [MMA]_0_:[EBPA]_0_:[Fe(acac)_3_]_0_:[PPh_3_]_0:_[ACHN]_0_, *V*_toluene_ = 1.0 mL, m(Fe(acac)_3_) = 0.34 mg, T = 90 °C, polymerization time = 2.5 h; ^a^ *M*_n,th_ = ([M]_0_/[I]_0_) × *M*_n,MMA_ × conv.%.

### 3.6. Polymerization Mechanism and Polymerization Kinetics

**Scheme 1:**
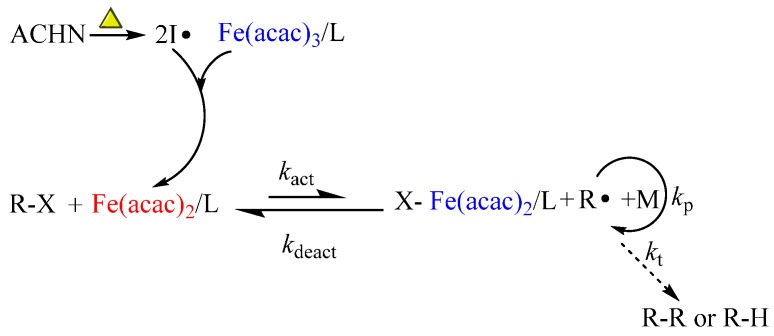
Possible polymerization mechanism of ICAR ATRP with Fe(acac)_3_ as the initial catalyst and ACHN as the azo-initiator.

The possible polymerization mechanism of iron(III)-mediated ICAR ATRP is shown in Scheme 1. We assume that, in the beginning, radicals produced by the azo initiator (ACHN) reduce Fe(acac)_3_ to Fe(acac)_2_, even though the mechanism behind this reduction is not yet fully understood. Then, the generated active Fe(II) species can seize the halogen from the ATRP initiator (R–X) and form the X–Fe(acac)_2_/L as well as the propagating radical. The next steps are the same as our previous document [69]. The difference between ICAR ATRP and normal ATRP is that the azo-initiator (ACHN here) can produce free radicals constantly to activate the Fe(III) species to establish a dynamic equilibrium between the active Fe(II) species and Fe(III) species and therefore to mediate the concentration of propagating radicals to control the polymerization.

**Figure 1:**
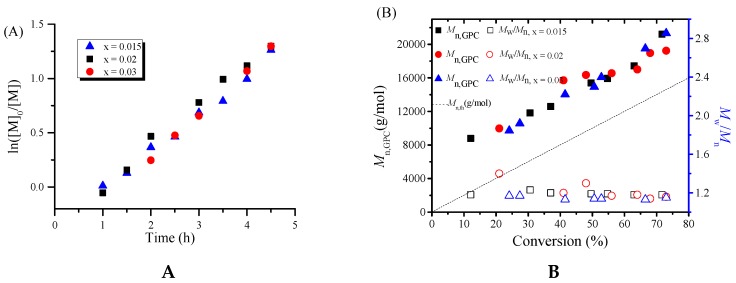
ln([M]_0_/[M]) as a function of time (**A**) and evolution of number-average molecular weight (*M*_n,GPC_) and molecular weight distribution (*M*_w_/*M*_n_) versus conversion (**B**) for ICAR ATRP of MMA with various iron catalyst concentration. Polymerization conditions: [MMA]_0_:[EBPA]_0_:[Fe(acac)_3_]_0_:[PPh_3_]_0_:[ACHN]_0_ = 200:1:*x*:0.3:1 (*x* = 0.015, 0.02, 0.03), *V*_MMA_ = 1.0 mL, *V*_toluene_ = 1.0 mL, T = 90 °C.

**Figure 2:**
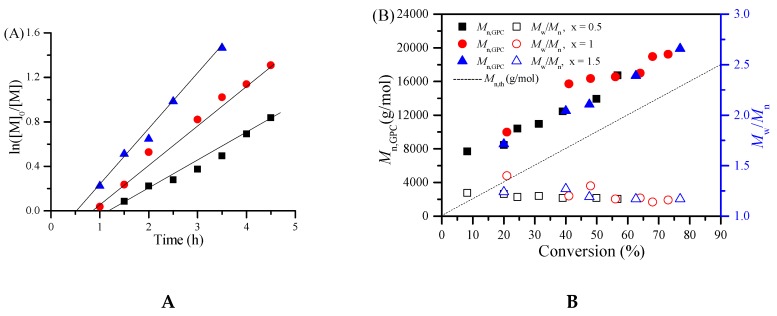
ln([M]_0_/[M]) as a function of time (**A**) and evolution of number-average molecular weight (*M*_n,GPC_) and molecular weight distribution (*M*_w_/*M*_n_) versus conversion (**B**) for ICAR ATRP of MMA with various ACHN concentration. Polymerization conditions: [MMA]_0_:[EBPA]_0_:[Fe(acac)_3_]_0_:[PPh_3_]_0_:[ACHN]_0_ = 200:1:0.02:0.3:*x* (*x* = 0.5, 1, 1.5), *V*_MMA_ = 1.0 mL, *V*_toluene_ = 1.0 mL, T = 90 °C.

In order to further investigate the detailed polymerization behaviors, the polymerization kinetics of MMA were conducted with the molar ratio of [MMA]_0_:[EBPA]_0_:[PPh_3_]_0_:[ACHN]_0_ = 200:1:0.3:1 under various concentration of iron catalyst firstly. Figure 1A shows the kinetic plots of ln ([M]_0_/[M]) versus time. We can see that the polymerization was approximately first order with respect to the monomer concentration, indicating that the propagating radicals remained almost constant during the polymerization in the three cases. From Figure 1B, it can be seen that the molecular weights increased linearly with monomer conversion while keeping narrow molecular weight distributions (*M*_w_/*M*_n_ ≤ 1.3). Furthermore, we also studied the polymerization kinetics under different concentration of azo-initiator ACHN. As is shown in Figure 2A, the polymerization was basically first order with respect to the monomer concentration. Moreover, the increase of the amount of ACHN can shorten the induction period of the polymerization system and accelerate the polymerization rate at the same time. Similarly, from Figure 2B, the linear increase of the molecular weights with monomer conversion and narrow molecular weight distributions can also observed. These polymerization kinetics further demonstrated the “living” features of this iron-mediated homogeneous ICAR ATRP system using Fe(acac)_3_ as the catalyst and ACHN as the azo-initiator.

### 3.7. Analysis of Chain-End and Chain Extension

**Figure 3:**
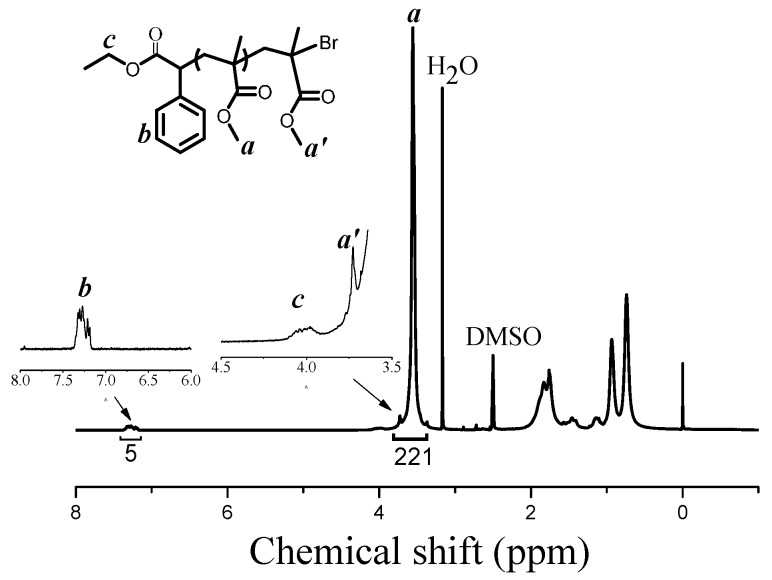
^1^H NMR spectrum of the resultant PMMA with DMSO-d_6_ as solvent and tetramethylsilane (TMS) as internal standard. Polymerization conditions of sample PMMA (*M*_n,GPC_ = 6570 g/mol, *M*_w_/*M*_n_ = 1.16): [MMA]_0_:[EBPA]_0_:[Fe(acac)_3_]_0_:[PPh_3_]_0_:[ACHN]_0_ = 200:2:0.03:0.3:1, *V*_MMA_ = 2.0 mL, *V*_toluene_ = 1.0 mL, T = 90 °C, polymerization time = 1.5 h.

**Figure 4:**
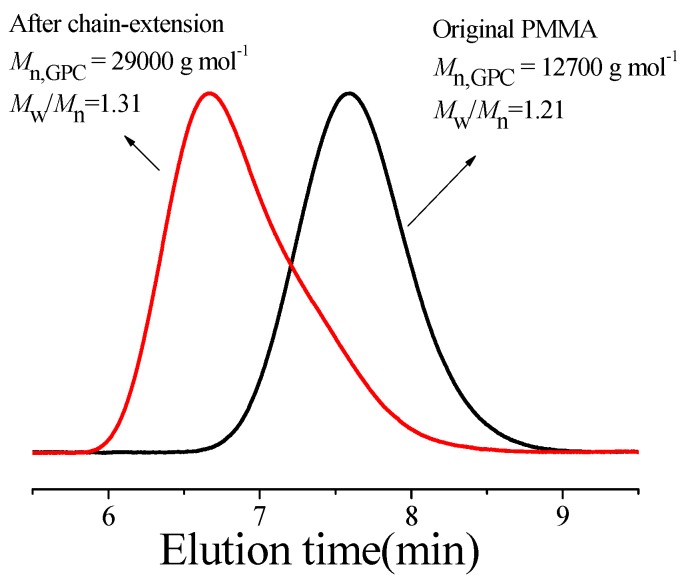
Gel permeation chromatograph (GPC) traces of PMMA before and after chain extension. Chain extension polymerization conditions: [MMA]_0_:[PMMA]_0_:[Fe(acac)_3_]_0_:[PPh_3_]_0_:[ACHN]_0_ = 200:1:1:3:0.75, *V*_MMA_ = 0.2 mL, *V*_toluene_ = 1.0 mL, T = 90 °C, polymerization time = 18 h.

The chain-end of the resultant PMMA was analyzed by ^1^H NMR spectroscopy. The signals at δ = 4.0 ppm (***c*** in Figure 3) and 7.3 ppm (***b*** in Figure 3) were assigned to the methylene and phenyl groups in the initiator EBPA, respectively, which indicated the initiator EBPA moieties were attached to the polymer chain-end successfully. The signal at δ = 3.60 ppm (***a*** in Figure 3) corresponded to methyl ester groups in PMMA. The signal at δ = 3.78 ppm (***a^’^*** in Figure 3) was assigned to methyl ester group at the chain-end [98,99]. Further, we conducted the chain-extension experiment using the resultant PMMA as the macroinitiator (*M*_n,GPC_ = 12,700 g/mol, *M*_w_/*M*_n_ = 1.21), Fe(acac)_3_ as the catalyst and ACHN as the azo-initiator with fresh MMA. As is shown in Figure 4, the *M*_n,GPC_ increased to 29,000 g/mol (*M*_w_/*M*_n_ = 1.31) after chain extension. Based on the above phenomenon, we can draw the conclusion that polymeric product has a high degree of chain-end functionality, further verifying the “living” features of this polymerization system. However, the GPC trace of PMMA after chain-extension showed a little shoulder, which indicated that the high degree of chain-end functionality was somewhat limited. This may be caused by the use of a relatively large amount of thermal initiator ACHN relative to alkyl halide.

## 4. Conclusions

A facile iron-mediated homogeneous ICAR ATRP system was developed using organometallic catalyst Fe(acac)_3_ and azo-initiator ACHN successfully in toluene. The polymerization of MMA can be carried out smoothly even if the amount of the iron catalyst decreases as low as to 1 ppm, fully demonstrating high activity of the iron catalytic system.
